# Supplementary figures and images for: The evidence for anthocyanins in the betalain-pigmented genus Hylocereus is weak
Source: BMC Genomics. 2022 Nov 9;23:739. doi: 10.1186/s12864-022-08947-1 (PMC9644512; doi:10.1186/s12864-022-08947-1)

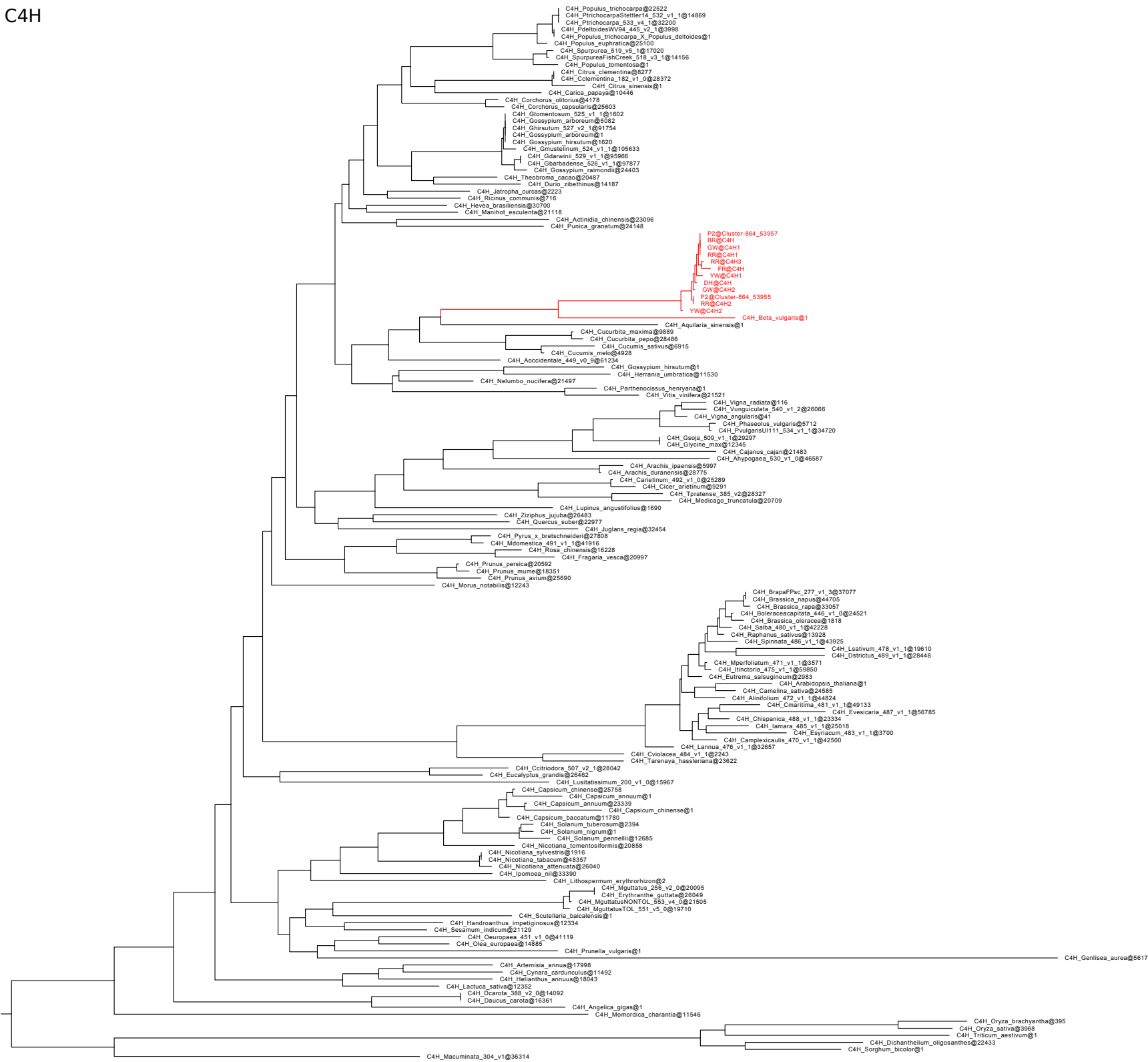

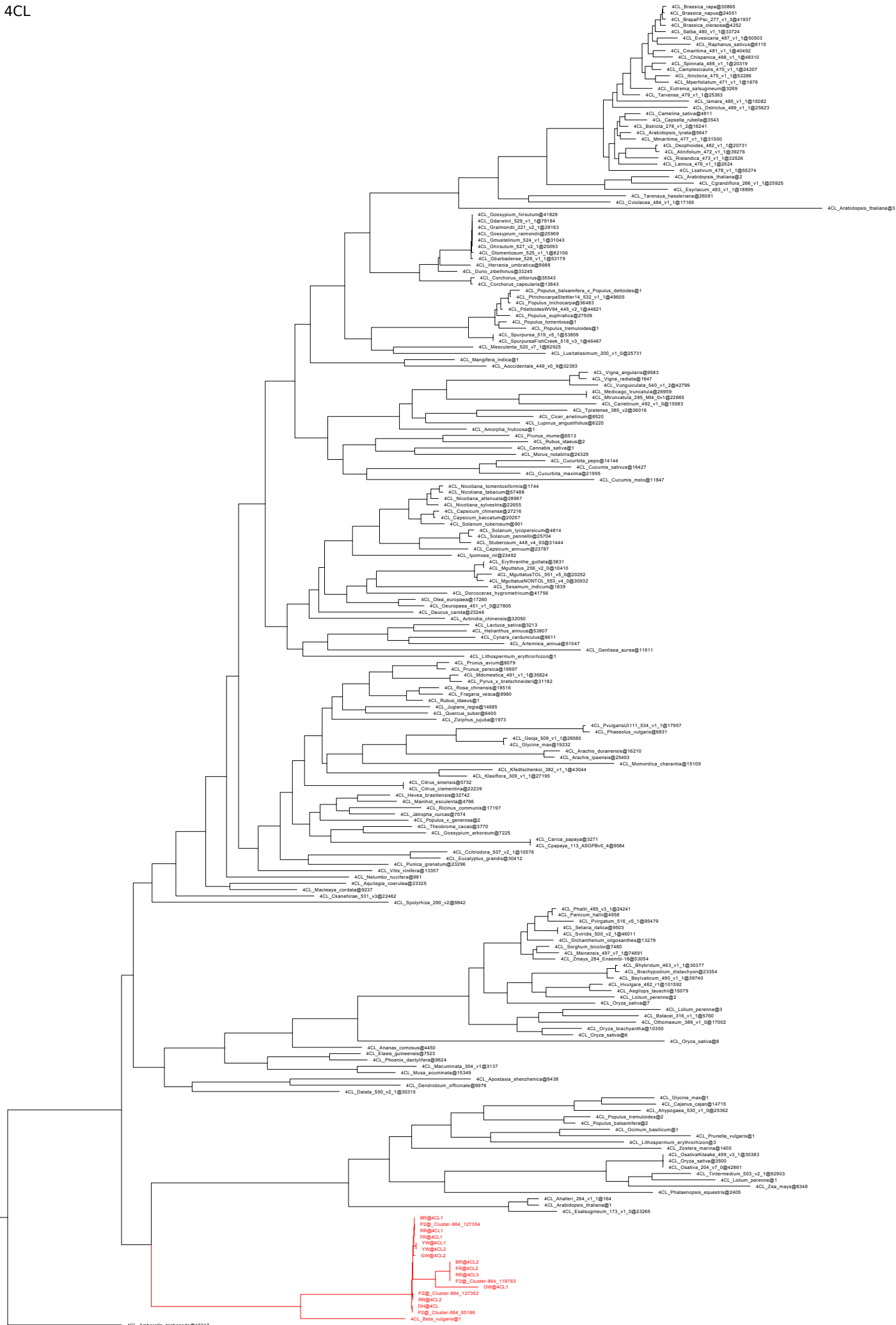

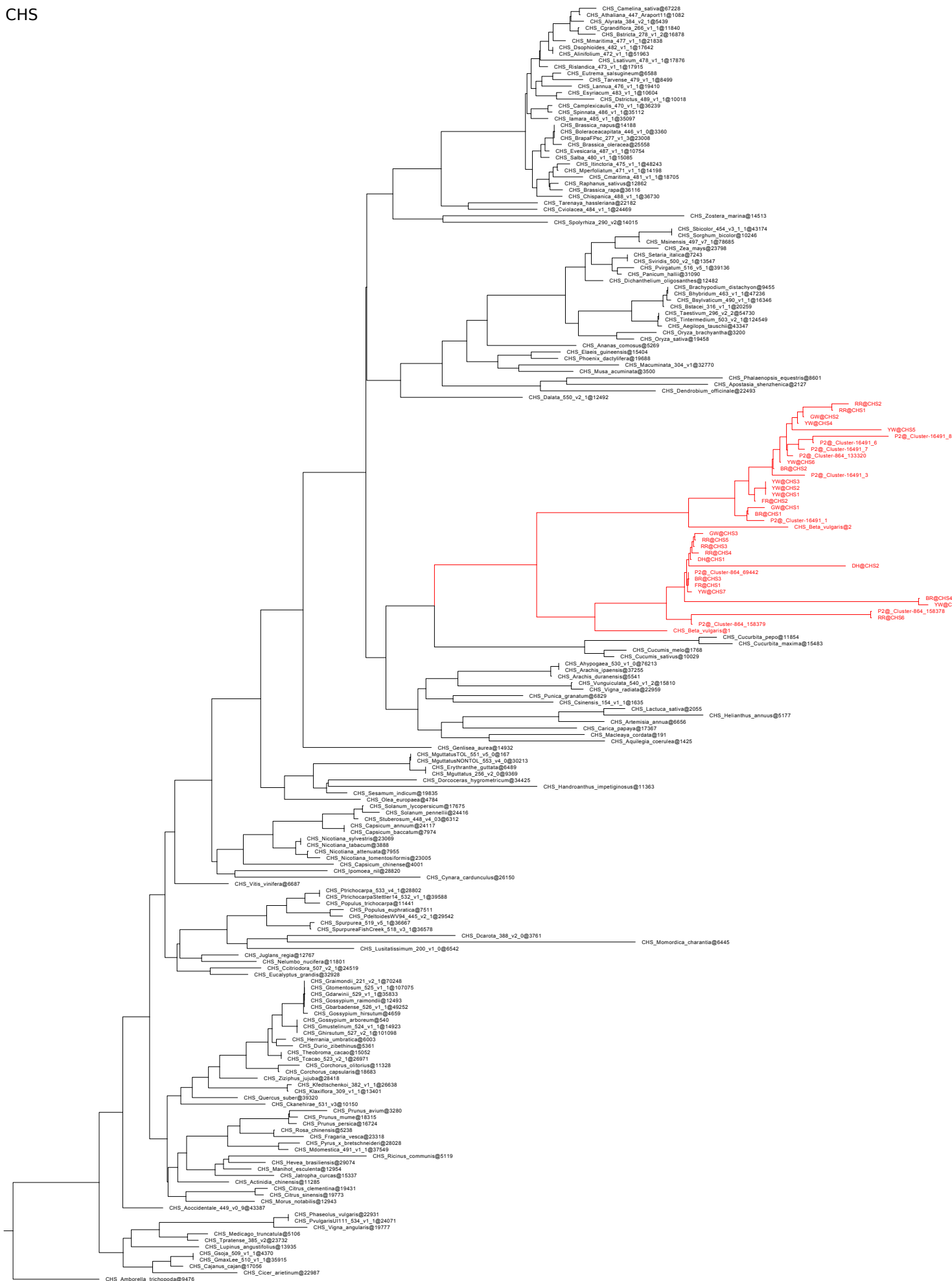

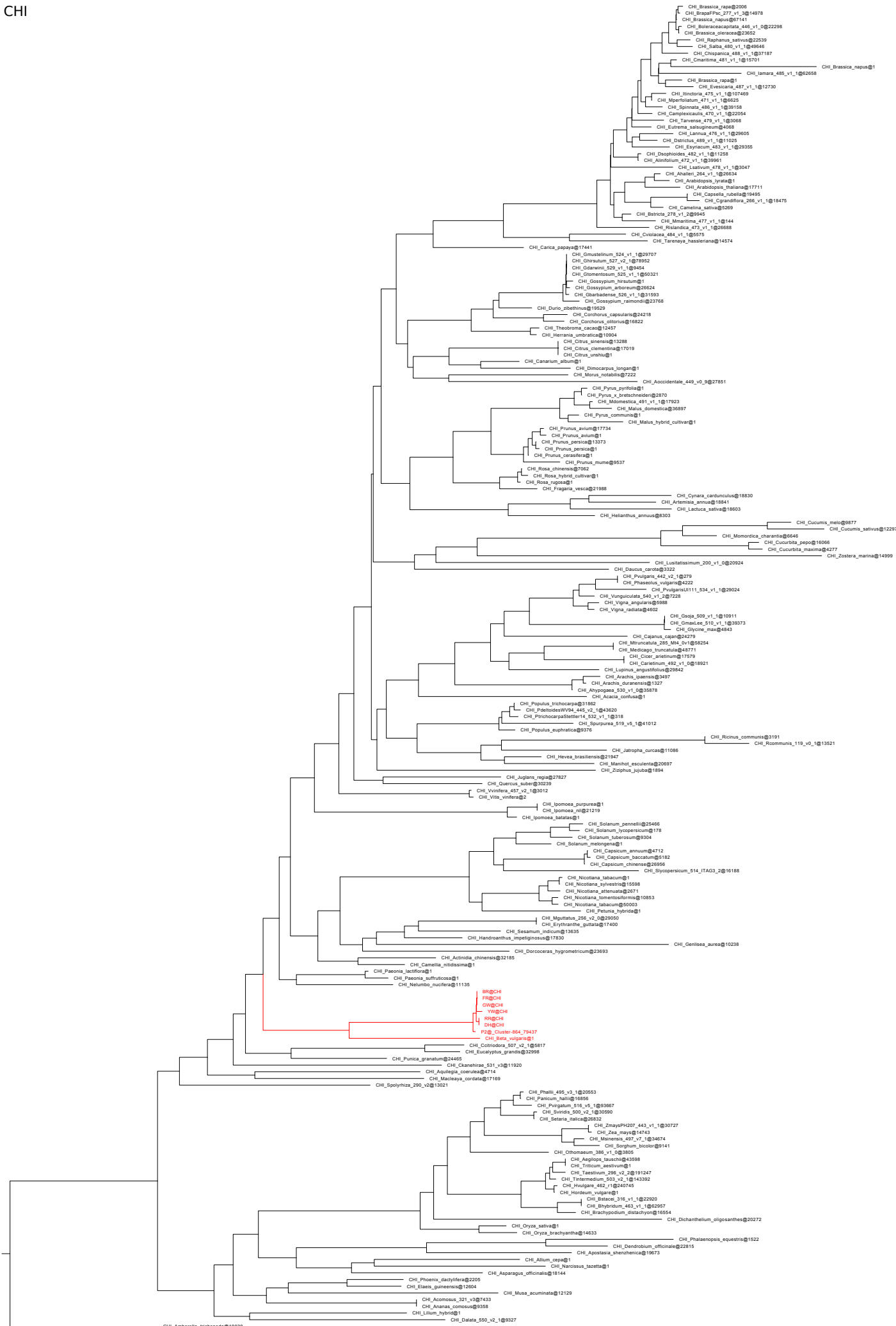

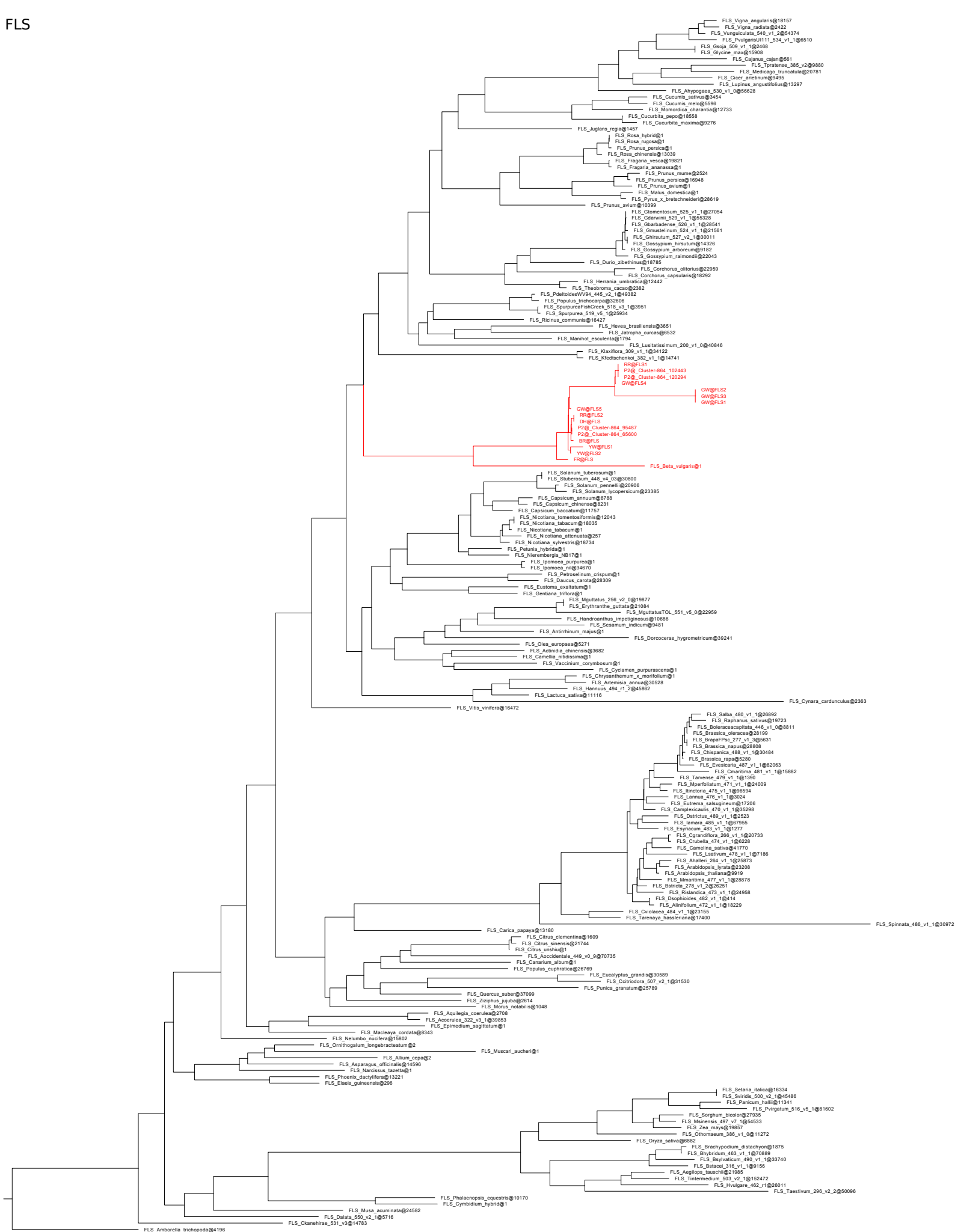

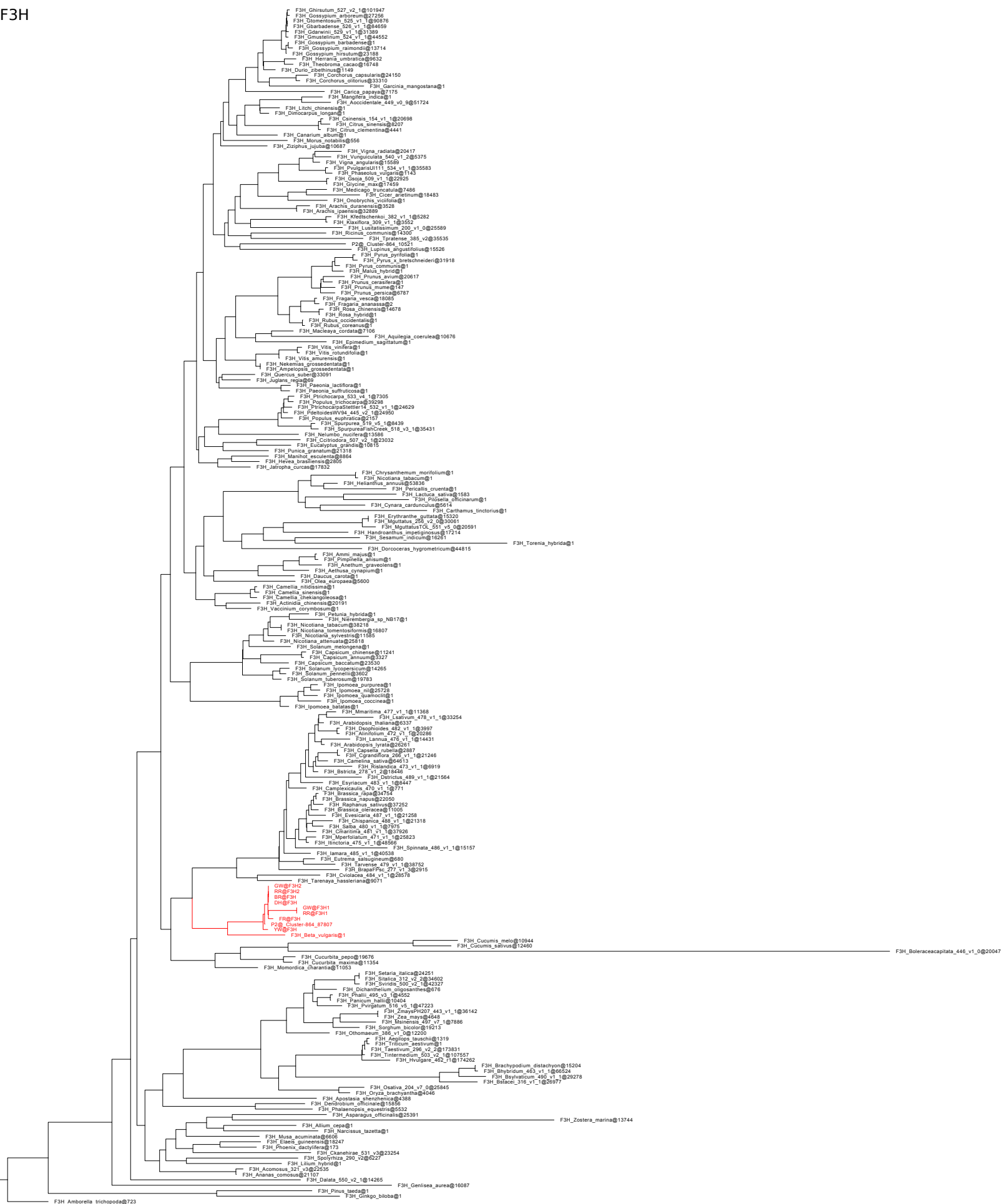

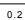

## DFR

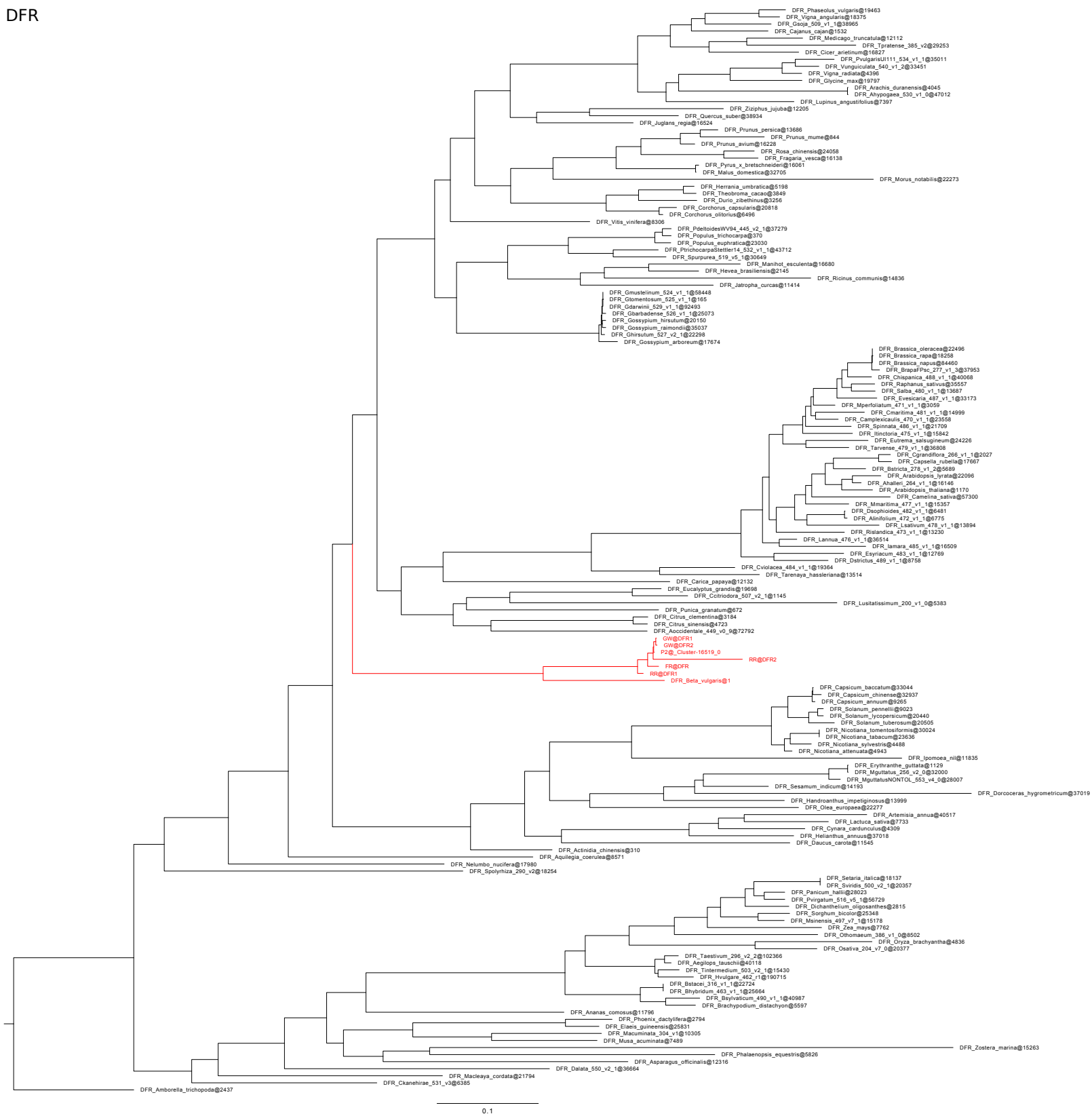

ANS

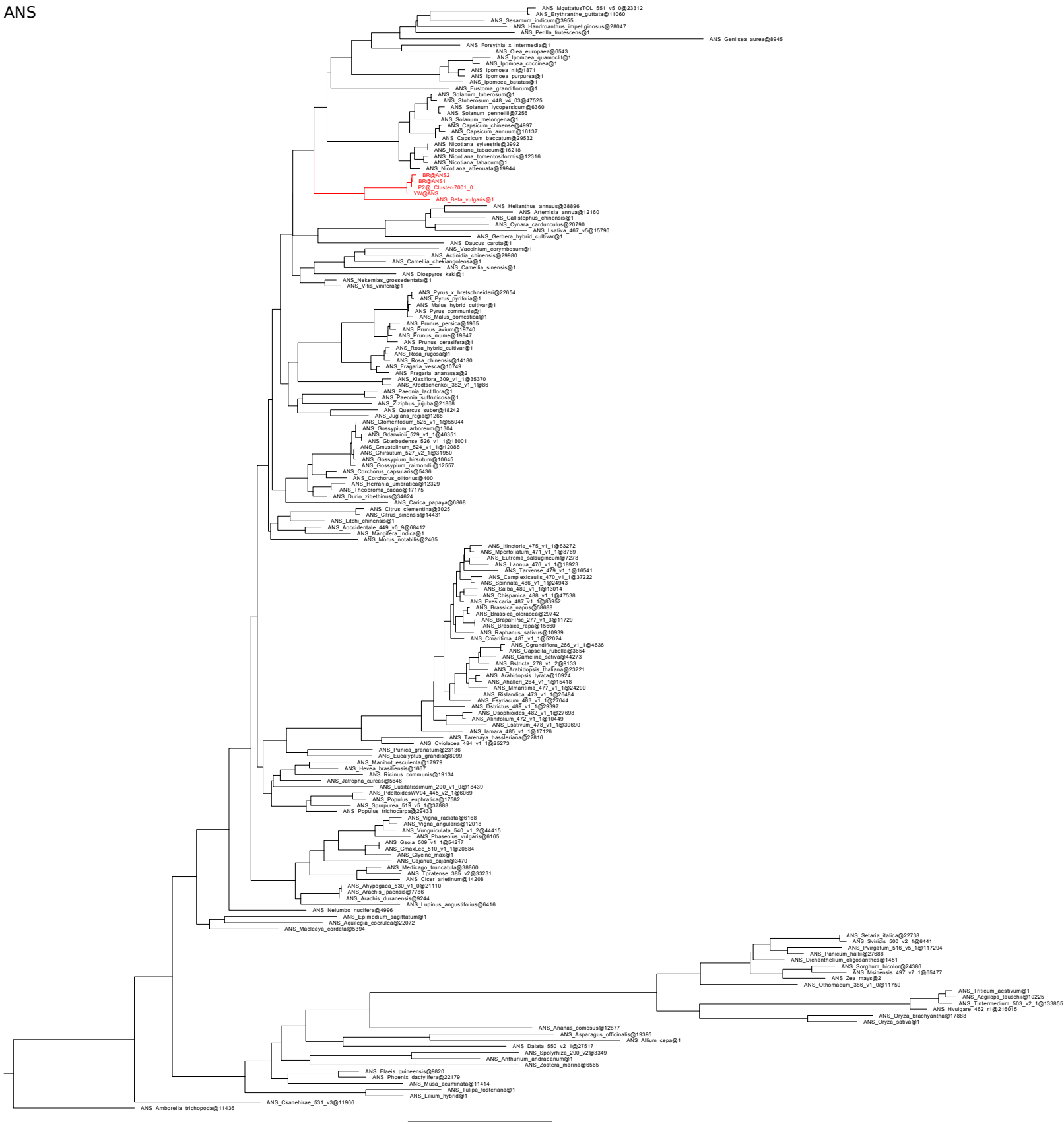

LAR

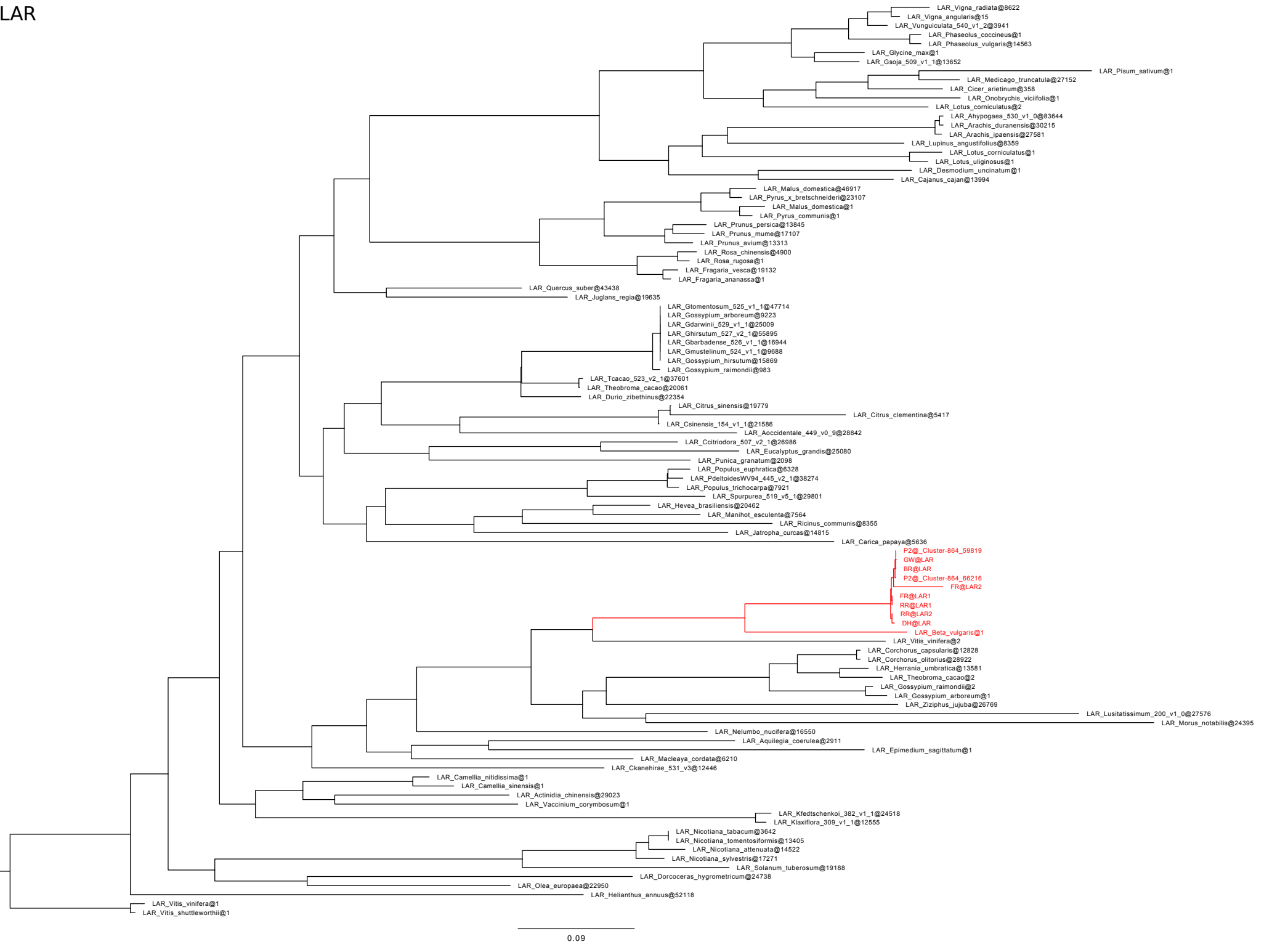

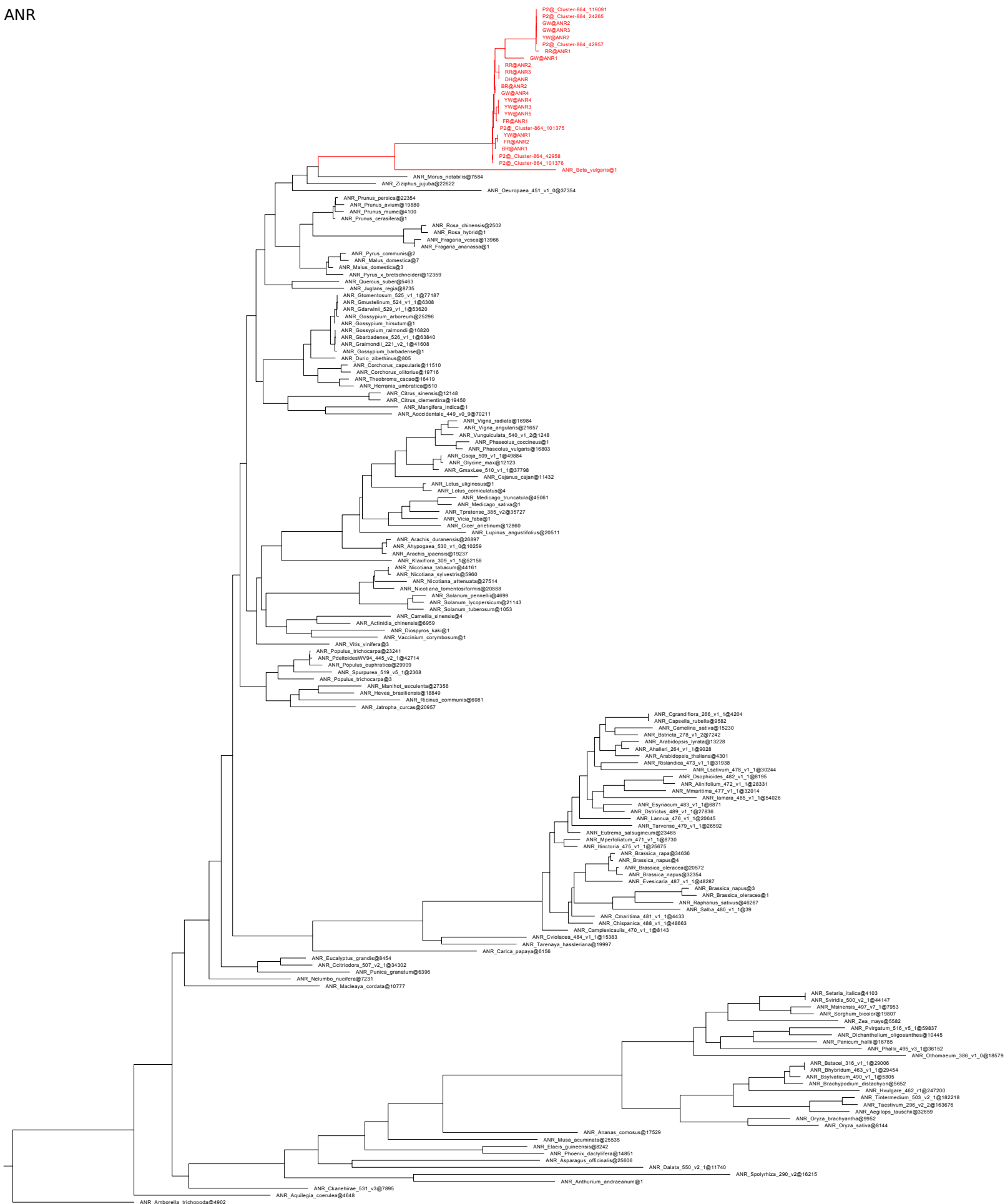

Supplement: Supplementary file 1 — Additional file 1. Phylogenetic trees of genes in the flavonoid biosynthesis. Identified candidate sequences are highlighted in red. [file 12864_2022_8947_MOESM1_ESM.pdf]
